# Postpartum Depressive Symptoms and Their Selected Psychological Predictors in Breast-, Mixed and Formula-Feeding Mothers

**DOI:** 10.3389/fpsyt.2022.813469

**Published:** 2022-02-02

**Authors:** Karolina Kossakowska, Eleonora Bielawska-Batorowicz

**Affiliations:** Department of Clinical Psychology and Psychopathology, Faculty of Educational Sciences, Institute of Psychology, University of Lodz, Lodz, Poland

**Keywords:** postpartum depression, feeding methods, feeding beliefs, feeding behaviors, maternal competencies, stress, mother-child bonding disorders

## Abstract

**Background:**

Although breastfeeding is recommended by WHO and professionals as the most beneficial for newborn babies, many women find it challenging. Previous research yielded ambiguous results concerning the role of breastfeeding in the development of postpartum depression. The study aimed to identify the best predictors of depressive symptoms for each of these feeding method.

**Methods:**

The participants were 151 women (mean age 29.4 yrs; SD = 4.5) who gave birth within the last 6 months and included 82 women classified as breastfeeding, 38 classified as mixed-feeding (breast and bottle), and 31 as formula-feeding. The study had a cross-sectional design using a web-based survey for data collection. The following measures were administered: The Edinburgh Postnatal Depression Scale; Sense of Stress Questionnaire; The Postpartum Bonding Questionnaire; Parenting Sense of Competence Scale; Infant Feeding Questionnaire.

**Results:**

Women in study groups differed in stress, bonding difficulties, and beliefs related to feeding practices and infancy. There were no significant differences in the severity of depressive symptoms, but all mean EPDS scores were above 12. Maternal satisfaction, intrapsychic stress, and concerns about feeding on a schedule were the best predictors of EPDS scores for breastfeeding women. For mixed-feeding – emotional tension, concern about infant's hunger, overeating, and awareness of infant's hunger and satiety cues; while for the formula-feeding group, predictors included emotional tension, bonding difficulties, and such maternal feeding practices and beliefs as concern about undereating, awareness of infant's hunger and satiety cues, concerns about feeding on a schedule and social interaction with the infant during feeding.

**Conclusion:**

Differences in predictors of postpartum depression for study groups suggest that breastfeeding itself may not be a risk for postpartum depression. However, the specificity of maternal experiences with the various types of feeding is related to difficulties promoting postpartum depression. Providing emotional and educational support appropriate for different types of feeding may be an essential protective factor for postnatal depression.

## Introduction

Breastfeeding is strongly advised for at least the first 6 months of a child's life. It is advocated for its benefits for infants' health and emotional development (1). Breastfeeding is also considered the main maternal task, and women are expected to choose it and continue as long as possible. A variety of strategies used to promote breastfeeding include those concentrated on the benefits of such a feeding method and those focused on the risk of formula feeding (2). Initiation of breastfeeding usually takes place in postnatal wards. In this process, the support from medical staff plays an important role (3). Despite benefits for children, breastfeeding is also analyzed in the context of its effects for mothers, especially the risk of postpartum depression.

Postpartum depression (PPD) is considered a public health issue. It might affect as many as 9.6% of new mothers in high-income and 19.6% in low-income countries (4). Among factors associated with PPD, breastfeeding is often considered. However, its role either as a risk or as a protective factor is debated. Earlier studies indicated that exclusive breastfeeding increased the risk of PPD (5, 6) and that postpartum depression was more common among breastfeeding mothers (7). Such a view was not universal, as findings from other studies indicated the opposite – there were more cases of PPD among bottle-feeding mothers (8). More recent studies examining the effect of breastfeeding on PPD revealed a different pattern. According to Toledo et al. (9) and Gila-Diaz et al. (10), women who currently breastfed their infants or breastfed them for a more extended time expressed significantly lower PPD risk. In line with this finding are the results from the study by Islam et al. (11) that have pointed to the role of early cessation of exclusive breastfeeding for the increased risk of PPD. The mixed-feeding method might also be related to increased depression symptoms postpartum (12, 13). However, such results are not universal, as Fukui et al. (14) have found that breastfeeding did not affect PPD. The lack of clear breastfeeding – PPD link was also confirmed in the systematic review and meta-analysis conducted by Woldeyohannes et al. (15).

The relationship between breastfeeding and postpartum depression seems well documented, although the type of such association – whether breastfeeding decreases or increases the PPD risk – is still debated. The inclusion of additional factors might help in such a debate. One of such factors that might moderate the association of PPD and infant feeding is maternal breastfeeding self-efficacy. Its low level was associated with higher PPD scores in studies by Zubaran and Foresti (16) and Kossakowska (17). Another factor was maternal positive breastfeeding attitude associated with lower depressive symptoms at 6 months postpartum (18). Yet another factor was the satisfaction with breastfeeding – it was higher in women without PPD symptoms (19). Thus the relationship between breastfeeding and postpartum depression was analyzed in the context of various aspects of breastfeeding (i.e. duration, self-efficacy, positive/negative experiences, or attitudes toward breastfeeding) rather than in the context of the type of feeding itself. It is unclear whether the same factors are related to PPD symptoms for mothers who apply either of three feeding methods: breastfeeding, mixed, or formula feeding. Our study aimed to clarify whether mothers were at similar risk of postpartum depressive symptoms for each of these feeding methods. As the benefits of breastfeeding for maternal bonding were advocated for, we aimed to verify whether the mother-child bond developed differently in either of three feeding methods and whether PPD was similarly connected to maternal feeding beliefs, maternal self-esteem, and stress. Thus we aimed to identify the best predictors of depressive symptoms for each of these feeding methods.

## Materials and Methods

### Study Design

This descriptive web-based cross-sectional study was conducted to identify predictors of postpartum depression for different feeding methods.

### Ethical Consideration

The research procedure was performed in accordance with the Helsinki Declaration of Human Rights (20). The study was approved by the university advisory board. As the study was of an informative cross-sectional purely descriptive nature, no formal ethical approval was required under the country's legislation. Participants were informed of the purpose, risks, and benefits of the survey. They were told they could withdraw from the study at any time and for any reason and provided electronic informed consent. Such consent form was prepared following the Ethics Guidelines for Internet Mediated Research (21).

### Inclusion Criteria

The following inclusion criteria were applied: at least 19 years of age at the time of admission to the study, giving birth to a healthy child within the last 6 months^1^, no past or current clinical diagnosis of any psychiatric disease, including depression. As our study aimed to identify risk factors for postpartum depression depending on whether women are exclusively breastfeeding, mixed or formula milk only, the classification criteria for each of these groups were based on the WHO-recommended definitions. Exclusive breastfeeding was defined as infants being fed with breast milk only, without any additional food or drink, not even water (allowable exceptions were expressed breast milk, oral rehydration solutions, and drops or syrups of vitamins and minerals and medicines) (22). Mixed-feeding (MF) suggested that infants were fed breast milk with formula or complementary food. Formula-feeding (FF) was defined as infants being fed with any formula (23).

### Procedure and Data Collection

The presented data were collected from July 2017 to March 2018. Women were recruited through social media (such as Facebook and Instagram) advertisements, information distributed at birth classes or pediatric clinics, and snowball sampling of participants' friends and relatives. Women interested in participating in the study first contacted the researcher by e-mail (the e-mail address was given in the recruitment advertisement). They had to agree to participate by signing an electronic informed consent form. After that, they received a personalized link to the web-based survey.

Initially, a total of 187 mothers were interested in participating in the study. Of these, 31 were rejected at the recruitment stage due to failure to meet the inclusion criteria (i.e. more than 6 months from childbirth, younger than 19 years of age). Of the remaining 156 volunteers, five women did not fully complete questionnaires. Finally, results from 151 mothers (BF: *n* = 82; MF: *n* = 38; FF: *n* = 31) who met the eligibility criteria were included in the analyses.

### Measures

#### Sociodemographic and Perinatal Questionnaire

Sociodemographic and perinatal characteristics included maternal age, level of education, financial status, relationship status, personal health history, number of weeks from childbirth, course of pregnancy, childbirth, and feeding experiences.

#### Postpartum Depression Symptoms

The Edinburgh Postnatal Depression Scale (EPDS) (24) assessed depressive symptoms among participants. EPDS is a well-validated 10-item self-report scale constructed to measure the intensity of depressive symptoms within the last seven days. Each item is rated on a 4-point scale ranging from 0 to 3. The higher scores indicate greater symptom severity (the Authors recommend a 12/13 cut-off point). Original research reports good internal consistency (24) – Cronbach's alpha = 0.87, which was confirmed in the validation study of the Polish version of EPDS (Cronbach's alpha = 0.91) (25). In the present sample, Cronbach's alpha was 0.83.

#### Stress Level

Sense of Stress Questionnaire (KPS – Kwestionariusz Postrzeganego Stresu) (26) measured the experienced stress. The questionnaire allows assessing total stress level and its three dimensions: emotional tension, external stress, and intrapsychic stress. KPS is a 27-items self-report measure. Each item is rated on a 5-point scale ranging from 1 (false) to 5 (true). The authors of the questionnaire reported high internal consistency for all scales, ranging from 0.70 to 0.81. In our sample, Cronbach's alpha value for total scores was 0.93.

#### Mother-Child Bond

The Postpartum Bonding Questionnaire (PBQ) (27) was used to assess the bond between mother and baby. The PBQ is a 25-item self-report instrument. In our study, we used the pre-validated Polish language version of the questionnaire (Bieleninik, unpublished materials). Each item of PBQ is rated on a 6-point scale ranging from 0 to 5. It consists of four subscales: impaired bonding (scale 1), rejection and anger (scale 2), anxiety about care (scale 3), and risk of abuse (scale 4). The total scores can also be calculated, and higher scores suggest poorer bonding. Reliability of PBQ in validation study was satisfactory - Cronbach's alpha for total scores was 0.80 (28, 29) and 0.92 in the current sample. In the present study, the risk of abuse scale results was not analyzed because it is a factor with only two items and the Cronbach's alpha was below the recommended value of 0.70.

#### Maternal Self-esteem

Parenting Sense of Competence Scale (PSOC) (30, 31) in Polish validated version (32) was used to examine maternal self-esteem on two dimensions – satisfaction and efficacy. PSOC is a self-report scale with 16 items assessed on a 6-point scale (ranging from 1 – strongly agree to 6 – strongly disagree). The satisfaction subscale refers to mothers' anxiety, motivation, and frustration, while the efficacy subscale assesses competence, problem-solving ability, and capability in the maternal role. Higher scores suggest higher competencies. The internal consistency of PSOC is satisfactory. The Cronbach's alpha coefficients for the total score were 0.79 in the original and 0.78 in the current study.

#### Maternal Feeding Behaviors and Beliefs

Infant Feeding Questionnaire (IFQ) (32) was used to identify maternal feeding practices and beliefs during infancy. IFQ is a 28-item self-report instrument. Items are rated on a 5-point scale, from 0 (never/disagree a lot) to 4 (always/agree a lot). In case of some statements (e.g. “I believed it was important for him to finish all of the formulae in his bottle,”) “not applicable” response was added to make IFQ suitable for exclusively breastfeeding mothers. The questionnaire allows to assess maternal feeding behaviors/practices and beliefs on seven dimensions: concern about infant undereating or becoming underweight (factor 1), concern about infant's hunger (factor 2), awareness of infant's hunger and satiety cues (factor 3), concern about infant overeating or becoming overweight (factor 4), feeding infant on a schedule (factor 5), using food to calm infant's fussiness (factor 6), social interaction with the infant during feeding (factor 7). Original development and validation study revealed internal consistency for seven factors from 0.24 to 0.74 (32). Internal consistency on all seven scales in our sample was slightly higher than reported in the original validation study and ranged from 0.41 to 0.74. IFQ was designed to identify maternal feeding behaviors and beliefs during the first 12 months of their children's lives related to children becoming overweight in the second year of life. In the current study, the questionnaire was used to compare feeding attitudes in different feeding type mothers and to assess their predictive role for the occurrence of symptoms of postpartum depression.

### Data Analysis

All statistical analyses were performed using the Statistical Package for the Social Sciences (SPSS) version 25.0 for Windows. Demographic characteristics were summarized as the mean (standard deviation, SD) for continuous variables and frequency counts (percentages) for categorical variables. The chi-square test was then used to estimate the significance of differences between mothers with different feeding practices. The Shapiro-Wilk test was used to check the normality of distributions for all analyzed variables. Due to the lack of normality of the distribution, the non-parametric Kruskal-Wallis test (with the Dunn's pairwise tests adjusted by the Bonferroni correction) was applied to compare more than two independent groups. Spearman's correlation coefficient was used to assess a possible association between all continuous variables. Finally, the multivariate linear regression with a backward-elimination approach was used to estimate the predictors of postpartum depression for each type of feeding. For each regression model presented, the VIF (Variance Inflation Factor) value and its tolerance to detect multicollinearity in the regression analysis were determined. A VIF of 1 indicates no predictors of collinearity. The higher the value of VIF, the more significant the correlation of the outcome variable with other variables. According to the recommendation of Vittinghoff et al. (33), it was assumed that the VIF of 10 and more is regarded as very high, indicating strong collinearity of the predictors. In this case, the analyzed model should be corrected. Less liberal assumptions indicate that a VIF value> 5 means moderate multicollinearity (34), which is a cause for concern. The VIF values for the EBF and MF mothers regression models were not greater than 5 (from 1.014 to 1.020 for EBF and 1.014 to 1.246 for the MF group). In the FF group, the VIF value for impaired bonding was 6.858, indicating moderate collinearity based on more restrictive criteria. Therefore, the value of the tolerance coefficient for VIF was checked, which, according to Hair et al. (35), indicates a problem with multicollinearity when it is less than 0.2. In the current study, the tolerance for VIF in each regression model was more than 0.2. Therefore, the impaired bonding predictor was left in the regression model for the FF group. The level of statistical significance for the study was set at *p* < 0.05.

## Results

### Study Sample Characteristics

One hundred and fifty-one women aged 19 to 41 years (M = 29.4; SD = 4.5) who gave birth within the last 6 months participated in this study. Infants aged between 2 and 24 weeks (M = 17.5; SD = 5.5). Eighty-two women were classified as exclusively breastfeeding (EBF group), 38 were classified as mixed-feeding (breast and bottle) (MF group), and 31 as fully formula-feeding (FF group). According to the Kruskal-Wallis test, there were no differences between feeding groups in terms of women's age (H_(2)_ = 0.134; p = 0.935) as well as infants' age (H_(2)_ = 0.678; p = 0.935). In the sample, most women were primiparous (59.6%), without previous miscarriages (76.2%), planned pregnancy (78.8%), without complication (74.8%). Most of the participants had skin-to-skin contact with their baby soon after delivery (78.1%), and most women planned to breastfeed before childbirth (90.7%). Chi-square tests indicated a significant difference between the feeding method groups only for the infant's gender (?22,151 = 8.533; *p* < 0.05). In the EBF group, male infants predominated (67.1%), while in the MF and FF group, there were more female infants (55.3 and 58.1%, respectively). Detailed demographical and obstetrics characteristic of the feeding sub-samples is presented in Table 1.

**Table 1:** Characteristic of the study sample by the type of feeding.

		**EBF**		**MF**		**FF**		***χ^**2**^* (df)**	***p* value**
		***n*** **=** **82**		***n*** **=** **38**		***n*** **=** **31**			
		***N***	***%***	***N***	***%***	***N***	***%***		
Place of residence								6.900 (4)	0.141
City over 500,000 residents	51	62.2	20	52.6	25	80.6			
City below 500,000 residents	18	22.0	10	26.3	5	16.1			
Countryside	13	15.9	8	21.1	1	3.2			
Education								2.247 (2)	0.325
Higher education	64	78.0	29	76.3	20	64.5			
Marital status								0.281 (2)	0.869
Married	67	81.7	30	78.9	26	83.9			
Assessment of the financial situation								0.670 (2)	0.175
Good/very good	77	93.9	35	92.1	30	96.8			
The number of pregnancies								3.040 (4)	0.551
First	46	56.1	22	57.9	22	71.0			
Second	26	31.7	12	31.6	5	16.1			
Third and more	10	12.2	4	10.5	4	12.9			
Was the pregnancy planned								1.084 (2)	0.582
Yes	65	79.3	28	73.7	26	83.9			
Infant's gender								**8.533 (2)**	0.014^*^
Male	55	67.1	17	44.7	13	41.9			
Previous miscarriages								1.048 (2)	0.592
No	65	79.3	27	71.1	23	74.2			
Complications in the last pregnancy								0.446 (2)	0.800
No	63	76.8	28	73.7	22	71.0			
Complications of the last childbirth								2.494 (2)	0.287
No	63	76.8	25	65.8	20	64.5			
Delivery mode								0.560 (2)	0.756
Natural childbirth	45	54.9	20	52.6	19	61.3			
Feeding plan planned before the baby was born								3.387 (4)	0.495
EBF	77	93.9	32	84.2	28	90.3			
MF	3	3.7	3	7.9	1	3.2			
FF	2	2.4	3	7.9	2	6.5			
Skin-to-skin contact immediately after delivery								2.588 (2)	0.274
Yes	67	81.7	30	78.9	21	67.7			

*EBF, exclusively breastfeeding; MF, mixed-feeding (both breast and bottle); FF, formula-feeding*.

* *indicate p < 0.05*.

### Postpartum Depression Symptoms

**Figure 1:**
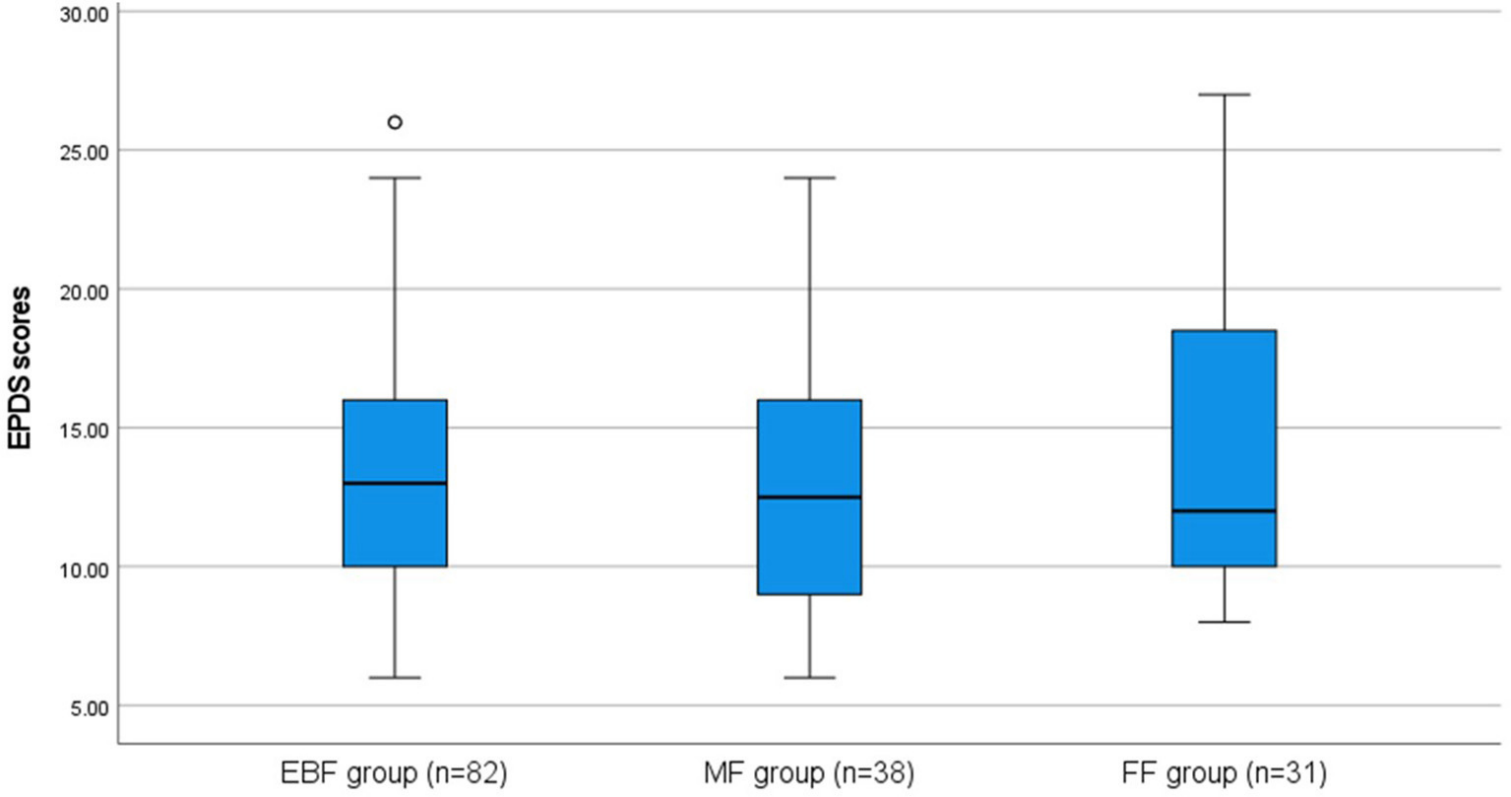
Range, means, and SD of EPDS scores by the feeding method.

The mean EPDS scores were 13.4 (SD = 4.2), 12.6 (SD = 4.1), 14.6 (SD = 5.4) for EBF, MF, and FF groups, respectively. There were no significant differences in the severity of postpartum depression symptoms between groups (H_(2)_ = 1.656; p = 0.437), but all mean EPDS scores were above 12 cut-off points. The range of EPDS scores for each group is shown in Figure 1.

### Stress Level, Parental Competencies, and Bonding

The scores for three groups of mothers calculated for the Sense of Stress Questionnaire (KPS), Postpartum Bonding Questionnaire (PBQ), Parenting Sense of Competence Scale (PSOC) as well as Infant Feeding Questionnaire (IFQ) are given in Table 2.

**Table 2:** Descriptive statistics for psychological variables analyzed in the study according to the feeding method.

	**EBF**			**MF**			**FF**		
	***n*** **=** **82**			***n*** **=** **38**			***n*** **=** **31**		
	**M (SD)**	**Skewness**	**Kurtosis**	**M (SD)**	**Skewness**	**Kurtosis**	**M (SD)**	**Skewness**	**Kurtosis**
Maternal Self-efficacy (PSOC)	33.3 (4.2)	−0.14	−0.82	35.3 (3.5)	−0.29	−0.29	33.6 (4.6)	−0.09	−0.86
Maternal satisfaction (PSOC)	30.5 (4.8)	−0.21	−0.43	30.4 (4.8)	0.15	0.15	28.4 (5.2)	0.28	1.04
Maternal competence (PSOC total)	63.9 (7.1)	−0.03	−0.06	65.8 (6.8)	0.08	0.08	62.0 (7.2)	0.71	−0.31
Emotional tension (KPS)	19.3 (6.8)	0.11	−0.73	20.8 (7.8)	0.06	−1.06	23.5 (8.1)	−0.57	−0.57
External stress (KPS	18.5 (5.1.)	0.39	−0.78	19.1 (6.0)	0.30	−1.08	20.7 (5.5)	0.14	−0.99
Intrapsychic stress (KPS)	15.4 (5.9)	0.69	−0.16	15.8 (6.6)	0.33	−1.32	19.6 (7.9)	0.41	−0.65
Stress level (KPS total)	53.2 (15.9)	0.37	−0.73	55.8 (19.4)	0.24	−1.21	63.9 (19.6)	−0.02	−0.74
Impaired bonding (PBQ)	10.8 (3.7)	0.35	−0.63	12.4 (6.6)	1.67	3.73	14.9 (7.9)	1.51	2.87
Rejection and anger (PBQ)	3.3 (2.6)	0.65	−0.32	5.1 (4.8)	1.27	1.57	7.7 (6.3)	1.19	1.00
Anxiety about care (PBQ)	6.3 (2.9)	1.17	2.12	6.3 (2.8)	0.78	0.60	7.0 (2.6)	0.71	1.15
Bonding difficulties (PBQ total)	29.9 (6.7)	0.35	−0.53	33.2 (12.4)	1.58	2.74	39.2 (14.9)	1.34	2.23
Concern about infant undereating (IFQ)	8.0 (2.4)	0.35	−0.79	8.1 (2.7)	0.23	−0.54	8.9 (2.9)	−0.36	−1.22
Concern about infant's hunger (IFQ)	5.4 (3.8)	1.55	0.98	4.7 (2.6)	1.51	1.56	5.3 (2.6)	1.29	1.25
Awareness of infant's hunger and satiety cues (IFQ)	6.3 (1.9)	0.43	−0.84	5.9 (1.7)	0.35	−1.29	6.7 (2.2)	0.34	−1.10
Concern about infant overeating (IFQ)	4.5 (1.7)	1.03	0.11	3.6 (1.1)	3.09	1.24	5.1 (1.7)	−0.05	−1.47
Feeding infant on a schedule (IFQ)	6.2 (0.9)	−0.04	4.35	6.5 (0.9)	2.27	5.03	7.3 (1.1)	−0.20	−0.86
Using food to calm infant's fussiness (IFQ)	5.5 (1.2)	−0.69	0.65	5.5 (1.2)	−0.11	0.26	4.8 (1.5)	−0.72	−1.01
Social interaction with the infant during feeding (IFQ)	7.9 (2.0)	−0.90	0.04	7.6 (2.0)	−0.35	−1.18	7.5 (1.8)	−0.85	1.35

*EBF, exclusively breastfeeding; MF, mixed-feeding (both breast and bottle); FF, formula-feeding*.

A Kruskal-Wallis test indicated differences between the mean ranks of Impaired bonding (H_(2)_ = 9.272; p = 0.010), rejection and anger (H_(2)_ = 15.116; p = 0.001) and total bonding difficulties scores (H_(2)_ = 11.033; p = 0.004) across the groups. Thus, Dunn's pairwise tests were carried out, and the evidence was found for differences between the EBF and FF groups on impaired bonding, rejection, and anger and on the total score (*p* < 0.01, *p* < 0.001, and *p* < 0.01, respectively; with the Bonferroni correction). The median of Impaired bonding scores for EBF mothers was 68.85 compared to 96.77 in the FF mothers. The median of rejection and anger scores for EBF mothers was 65.12 compared to 100.35 in the FF mothers. And the median of total bonding difficulties scores for EBF mothers was 67.72 compared to 98.32 in the FF mothers. There was no evidence for a difference between the other groups and Anxiety about care scores (H_(2)_ = 5.265; *p* = 0.072).

Similarly, the use of the Kruskal-Wallis test to compare the three dimensions of stress and overall stress level by feeding method showed that there is a difference between the mean ranks of the emotional tension (H_(2)_ = 7.201; *p* = 0.027), intrapsychic stress (H_(2)_ = 7.063; *p* = 0.029) and total level of stress across the study groups (H_(2)_ = 6.963; *p* = 0.031). According to Dunn's pairwise tests, this difference exists between the EBF and FF groups in emotional tension (*p* < 0.05), intrapsychic stress (*p* < 0.05) and total stress level (*p* < 0.05).

The median score of emotional tension for EBF mothers was 68.69 compared to 93.27 for FF mothers. The median score of intrapsychic stress for EBF mothers was 70.55 compared to 94.50 for FF mothers. Finally, the overall stress level median score for EBF mothers was 69.44 compared to 93.76 for FF mothers. There was no evidence for a difference between the other groups and External stress (H_(2)_ = 3.407; *p* = 0.182).

There were no differences in maternal efficacy (H_(2)_ = 5.096; *p* = 0.078), maternal satisfaction (H_(2)_ = 5.658; *p* = 0.059), and overall competences (H_(2)_ = 5.048; *p* = 0.080) measured by PSOC among the study groups.

### Feeding Beliefs

According to data presented in Table 2 women differed in their beliefs related to feeding practices at infancy, but only in case of concern about infant overeating (H_(2)_ = 14.895; *p* < 0.01) and feeding infant on a schedule (H_(2)_ = 26.076; *p* < 0.001).

For the concern about infant overeating, the differences were found according to Dunn's pairwise tests between EBF and MF groups (*p* < 0.05) and between FF and MF groups (*p* < 0.001). The median of concern about overeating for the MF mothers was the lowest (Mdn = 56.75) in comparison to EBF (Mdn = 77.96) and FF mothers (Mdn = 99.68). There were no differences between breastfeeding and formula-feeding mothers.

For the Feeding infant on a schedule, Dunn's pairwise tests indicated the differences between FF and EBF (*p* < 0.001), as well as FF and MF groups (*p* < 0.01). The median of Feeding infant on a schedule for the FF mothers was the highest (Mdn = 106.47) in comparison to MF (Mdn = 72.91) and EBF mothers (Mdn = 65.91). There were no differences between breastfeeding and mixed-feeding mothers.

There were also no significant differences in other feeding beliefs such as concern about infant undereating or becoming underweight (H_(2)_ = 2.563; *p* = 0.278), concern about infant's hunger (H_(2)_ = 2.645; *p* = 0.266), awareness of infant's hunger and satiety cues (H_(2)_ = 2.147; *p* = 0.342), using feeding to calm infant's fussiness (H_(2)_ = 4.949; *p* = 0.084), and social interaction with the infant during feeding (H_(2)_ = 1.919; *p* = 0.383).

### Predictors of Postpartum Depression Among EBF, MF, and FF Groups

The multiple linear regression analysis was used to determine predictors of postpartum depression for each infant feeding method group. Before it was performed, Spearman's correlation analyses were conducted in the total sample to determine the relations between the variables considered for inclusion in regression analysis. Table 3 shows the relationships between the EPDS and the total scores and scores for each dimension of measured variables.

**Table 3:** Correlation of EPDS and other psychological variables scores (*N* = 151).

**Variable**	**Maternal self-efficacy (PSOC)**	**Maternal satisfaction (PSOC)**	**Maternal competence (PSOC total)**	**Emotional tension (KPS)**	**External stress (KPS)**	**Intrapsychic stress (KPS)**	**Stress level (KPS total)**	**Impaired bonding (PBQ)**	**Rejection and anger (PBQ)**	**Anxiety about care (PBQ)**	**Bonding difficulties (PBQ total)**	**Concern about infant undereating (IFQ)**	**Concern about infant's hunger (IFQ)**	**Awareness of infant's hunger and satiety cues (IFQ)**	**Concern about infant overeating (IFQ)**	**Feeding infant on a schedule (IFQ)**	**Using food to calm infant's fussiness (IFQ)**	**Social interaction with the infant during feeding (IFQ)**
Postpartum Depression (EPDS)	−0.28^*^	−0.50^*^	−0.48^*^	0.35^*^	0.36^*^	0.25^*^	0.36^*^	0.26^*^	0.28^*^	0.24^*^	0.29^*^	0.11	0.18^*^	0.22^*^	0.07	0.09	0.03	−0.04

* *p < 0.05*.

For the postpartum depression (EPDS scores), the strongest relationships were found between EPDS and maternal satisfaction (*rho* = −0.50; p < 0.01), and between EPDS and overall level of maternal competences (*rho* = −0.48; *p* < 0.01). As both correlation coefficients are negative, they indicate that a lower level of maternal satisfaction and overall level of maternal competencies are linked to higher intensity of postpartum depression symptoms.

EPDS scores were also positively but weakly correlated with all stress dimensions such as emotional tension (*rho* = 0.35; *p* < 0.01), external and intapsychic stress (*rho* = 0.36 and *rho* = 0.25, respectively; *p* < 0.01), and overall stress level (*rho* = 0.36; *p* < 0.01). That indicates that the higher stress is related to the higher intensity of postpartum depression symptoms.

A statistically significant but very weak positive correlation was also found between EPDS and impaired bonding (*rho* = 0.26; *p* < 0.01), rejection and anger (*rho* = 0.28; *p* < 0.01), anxiety about care (*rho* = 0.24; *p* < 0.01), and total bonding difficulties *(rho* = 0.29; *p* < 0.01), indicating that bonding difficulties were accompanied by higher intensity of postpartum depression symptoms. Among the behaviors and beliefs about infant feeding, only two were significantly related to EPDS scores: concern about infant's hunger (*rho* = 0.18; *p* < 0.05), and awareness of infant's hunger and satiety cues (*rho* = 0.22; *p* < 0.01). The results indicate that the more mothers were concerned about infant undereating and hunger and were aware of infant's hunger and satiety cues, the higher was the intensity of postpartum depression symptoms. However, these coefficients indicated a very weak correlation.

Multiple linear regression optimized by the backward-elimination method was conducted separately for each group of mothers to assess the predictors of postpartum depression (outcome variable). In all analyses, explanatory variables introduced into the regression equation included: maternal satisfaction, self-efficacy and overall level of maternal competencies, emotional tenses, external and intrapsychic stress and general stress level, impaired bonding, rejection, and anger, anxiety about care, and maternal feeding behaviors and beliefs such as concern about infant undereating or becoming underweight, concern about infant's hunger, awareness of infant's hunger and satiety cues, concern about infant overeating or becoming overweight, feeding infant on a schedule, using food to calm infant's fussiness, and social interaction with the infant during feeding, represented by the relevant scores from administered measures.

Based on regression analysis results, it was found that the model proposed to predict postpartum depression in the EBF group was proven significant (F_(3, 81)_ = 10.347; *p* < 0,001). Three variables: maternal satisfaction, intrapsychic stress, and belief in feeding infants on a schedule, were significant in this model (adjusted R^2^ = 0.257, *p* < 0.01), and they simultaneously can explain 25.7% of the variance. The results of regression analysis are presented in Table 4.

**Table 4:** The multiple regression analysis for variables predicting postpartum depression among exclusively breastfed mothers (EBF group).

**Variable in the equation**	***B***	***SE B***	**β**	***t***	***p-value* [LL; HL 95% CI]**	***VIF***
Maternal satisfaction (PSOC)	−0.353	0.098	−0.349	−3.608	0.001 [−0.549; −0.158]	1.018
Intrapsychic stress (KPS)	0.179	0.069	0.249	2.570	0.012 [0.040; 0.317]	1.020
Feeding infant on a schedule (IFQ)	−1.338	0.540	−0.239	−2.479	0.015 [−2.413; −0.263]	1.014

*B, non-standardized regression coefficients; SE B, non-standardized regression coefficients error; β, standardized regression coefficient*.

The significant regression analysis model proposed to predict postpartum depression in the MF group (Table 5) included such variables as emotional tension, mothers' concerns about infants' hunger, awareness of infant's hunger and satiety cues, and concern about infant overeating (F_(4, 37)_ = 28.259; *p* < 0,001). All these variables simultaneously explain 74.7% of variance of postpartum depression (adjusted R^2^ = 0.747, *p* < 0.01).

**Table 5:** The multiple regression analysis for variables predicting postpartum depression among mix-fed mothers (MF group).

**Variable in the equation**	***B***	***SE B***	**β**	***t***	***p-value* [LL; HL 95% CI]**	***VIF***
Emotional tension (KPS)	0.355	0.044	0.678	8.131	<0.001 [0.266; 0.444]	1.014
Concern about infant's hunger (IFQ)	0.692	0.134	0.435	5.159	<0.001 [0.419; 0.964]	1.037
Awareness of infant's hunger and satiety cues (IFQ)	0.542	0.227	0.220	2.386	0.023 [0.080; 1.004]	1.246
Concern about infant overeating (IFQ)	−0.859	0.327	−0.241	−2.623	0.013 [−1.525; −0.193]	1.238

*B, non-standardized regression coefficients; SE B, non-standardized regression coefficients error; β, standardized regression coefficient*.

In the regression analysis for the last group – FF mothers, it was found that the model proposed to predict postpartum depression was proven significant (F_(7, 30)_ = 18.391; *p* < 0,001). The model (Table 6) included seven variables that altogether explain 80.3 % of variance of postpartum depression (adjusted R^2^ = 0.803, *p* < 0.001). Thus, the predictors of postpartum depression in the FF group were maternal emotional tension, impaired bonding and anxiety of infant care, concern about infant undereating, awareness of infant's hunger and satiety cues, feeding infant on a schedule, and social interaction with the infant during feeding.

**Table 6:** The multiple regression analysis for variables predicting postpartum depression among formula-fed mothers (FF group).

**Variable in the equation**	***B***	***SE B***	**β**	***t***	***p-value* [LL; HL 95% CI]**	***VIF***
Emotional tension (KPS)	−0.551	0.098	−0.820	−5.628	0.001 [−0.753; −0.348]	3.220
Impaired bonding (PBQ)	−0.924	0.117	−1.675	−7.879	<0.001 [−1.166; −0.681]	6.858
Anxiety about care (PBQ)	1.125	0.224	0.682	5.022	<0.001 [0.662; 1.588]	2.795
Concern about infant undereating (IFQ)	1.639	0.219	0.893	7.467	<0.001 [1.158; 2.093]	2.171
Awareness of infant's hunger and satiety cues (IFQ)	1.743	0.264	0.700	6.603	<0.001 [1.927; 2.289]	1.707
Feeding infant on a schedule (IFQ)	1.980	0.441	0.389	4.491	<0.001 [1.068; 2.893]	1.136
Social interaction with the infant during feeding (IFQ)	−4.327	0.543	−1.441	−7.965	<0.001 [−5.451; −3.203]	4.967

*B, non-standardized regression coefficients; SE B, non-standardized regression coefficients error; β, standardized regression coefficient; VIF, value of variance inflation factor*.

The predictive values of the β coefficient for Emotional tension (β = −0.551) and Impaired bonding (β = −0.924) are negative, which indicates that the occurrence of postpartum depression symptoms is explained by the low level of emotional tension and the lack of difficulties in building a mother-infant bond. We refer to this result in the Discussion section.

## Discussion

The first aim of our study was to clarify whether, for each of the analyzed feeding methods, mothers were at similar risk of postpartum depressive symptoms. Our findings indicate that there were no significant differences in the severity of such symptoms. These results seem to differ from some of those published so far, which indicate that the infant feeding method may be related to a maternal mood where breastfeeding mothers are less depressed (9, 10) or formula feeding women have higher rates of depression than women who breastfeed (36). Islam et al. (11) analysis show that non-exclusively breastfeeding mothers were more likely to experience depressive symptoms than exclusively breastfeeding mothers. Similarly, in the study conducted by Takashori (37), there was a significant difference in the prevalence of alleviated EPDS scores between breastfeeding and non-breastfeeding mothers (2.5 and 19.4%, respectively). Our results contradict those findings and indicate that maternal depressive symptoms are not related to the feeding method. However, it should be noted that the relationship between postpartum depressive symptoms and breastfeeding (including exclusive breastfeeding) is ambiguous and might be considered reciprocal – as the experience of depressive symptoms might affect breastfeeding initiation and its duration adversely.

Moreover, the relationship between depressive symptoms and feeding methods is more profound when additional variables are taken into account, such as breastfeeding self-efficacy (17) or breastfeeding intention (38). The study by Bora et al. (38) also indicates a link between breastfeeding and maternal depression, that is mediated by feeding intention, i.e. mothers who planned to breastfeed and went on to do so were around 50% less likely to become depressed than mothers who had planned to, and did not breastfeed. In our study, the mean EPDS scores in all feeding-type groups were above 12, the cut-off point indicating the depression risk. Therefore, they seem to confirm that the causes of depressive symptoms in the postpartum period are more complex and should be searched for among other factors, than the type of feeding.

The previous study has shown that prolactin and oxytocin production during breastfeeding is associated with lower maternal stress levels and enhanced mother-infant bonding (39). Our results indicate that formula-feeding mothers also experience greater emotional tension and higher intrapsychic and general stress than women who breastfeed exclusively. Also, the most increased bonding difficulties (reflected in higher scores on impaired bonding and rejection and anger) occurred in the formula-feeding group, while the lowest was among exclusively breastfeeding women. However, without considering the other factors that may mediate or moderate between feeding patterns and bonding with the baby, our findings should be interpreted with care and caution. Such an approach is supported by Hairston et al. (40) study, indicating that mother-infant bonding is not associated with feeding type. It suggests that if the mother has no other difficulties or mental disorders, breastfeeding is neither a threat to developing a bond with the child nor a protective factor for this bond.

The maternal feeding behaviors and beliefs were only partially connected to how the infant was fed. In the formula-feeding group, concerns related to providing feeding on a schedule were the highest compared to the other groups, while they were the lowest in the exclusive breastfeeding group. However, what seems important is that there were no differences between the exclusively breastfeeding and mix-feeding groups. These results seem to suggest that breastfeeding might be a factor preventing excessive worrying about whether the baby is being fed regularly, and it seems understandable, as breastfeeding should be on-demand. Thus, if the baby signals their hunger and the baby's weight and height increase in line with developmental norms, the mother does not have to additionally control the feeding hours, which reduces the number of concerns about caring for the baby. Perhaps this is one of the factors related to breastfeeding that minimizes the risk of postpartum depressive symptoms in breastfeeding women. Another difference between the groups is maternal concern about infant overeating or becoming overweight. The weakest concerns about overeating occurred in the group of mix-feeding mothers, while the strongest fears were typical for formula-feeding mothers. Similarly, as above, there were no differences between the exclusively breastfeeding and mix-feeding groups. Assuming that the fear of overeating in formula-feeding mothers may be accompanied, on the one hand, by the desire to strictly “stick to the feeding schedule” to ensure the child's proper physical development, and on the other hand, doubts as to whether the feeding times set by the schedule correspond to the baby's hunger, this may also be an explanation of the higher severity of depression among women who feed their children with formula milk.

Our findings concerning identifying the best predictors of depressive symptoms for each feeding method clearly indicate that there are differences in each group of mothers.

A lack of maternal satisfaction emerged as the most crucial predictor of mothers' EPDS scores in regression analysis for exclusively breastfeeding mothers. Although included in the regression equation, maternal satisfaction was previously not differentiated by the type of feeding. Previous studies have indicated a negative correlation between maternal satisfaction and postpartum depression (31, 41, 42). Similarly, in our study negative correlation between those variables was found as well. It may indicate that satisfaction or dissatisfaction associated with maternal role is primarily related to breastfeeding. Possible feeding failures may lower maternal satisfaction and thus increase the risk of depressive symptoms.

Moreover, the recent findings by Avilla et al. (19) indicate a positive association between maternal satisfaction with breastfeeding and PPD symptoms. In the current study, maternal satisfaction in general meaning was measured. However, since its association with postpartum depression did not appear anywhere except in the regression equation for the EBF group, mothers in this group likely assessed their satisfaction through the prism of breastfeeding. Two other predictors of depressive symptoms in this group are intrapsychic stress and concerns about feeding on a schedule. It should be clarified here that intrapsychic stress refers to the result of a woman's confrontation with herself as a mother. Thus, a high level of intrapsychic stress describes experiencing fears, worries, and a sense of losing meaning in life resulting from difficulties in overcoming the challenges of everyday life and achieving goals, tasks, and plans. Adaptation to motherhood, especially in its initial period, is often accompanied by difficulties in implementing tasks resulting from the new role, which may contribute to the occurrence of depressive symptoms. In addition, breastfeeding mothers who experience potential failure (e.g., due to inexperience in breastfeeding or difficulty in latching on to the breast) may be worried about their baby being provided with enough milk, which fosters depression.

In the group of mixed breastfeeding mothers, emotional tension was found among the predictors of postpartum depression. Further factors are related only to maternal concerns and behaviors associated with the course of feedings, such as concern about infant's hunger, awareness of infant's hunger and satiety cues, and concern about infant overeating. It appears that combining breastfeeding with formula milk may be associated with psychological benefits for mothers. On the one hand, while breastfeeding, they do not experience a feeling of failure as a mother, as they fulfill the social expectation for breastfeeding. Additionally, they share the special closeness that comes from physical contact with the baby during latching on to the breast, even if - as mentioned above - the influence of breastfeeding on the formation of the mother-infant bond is not as strong as it was supposed (40). On the other hand, formula milk gives a certain sense of security when feeding difficulties arise, or the mother gives up breastfeeding for personal reasons. At the same time, it should be noted that in this group - apart from emotional tension - the predictors of postpartum depression were factors related to the physiological aspects of a child's nutrition.

The highest number of predictive factors were found for formula-feeding mothers. Additionally, the direction of the indicated relationships is surprising. In univariate analysis, emotional tension and impaired bonding were positively correlated with maternal postpartum depression. Both emerged as the most important predictors of formula-feeding mothers' EPDS scores in regression analysis, and were negatively related to postpartum depression symptoms. It seems to us that the reasons for such results may be twofold. Firstly, univariate correlation analysis was carried out on the entire group, including all women participating in the study, regardless of the feeding method. Secondly, the role of fear of being judged by the environment should be considered. After having a baby, many women experience social pressure to breastfeed their babies. We assume that mothers nursing formula milk might fear ostracism and stigma when not breastfeeding. Thus, the disclosure of emotional tension and impaired bonding risks is being assessed even more negatively. This explanation seems to confirm the congruent direction of the relationship for another predictor of bonding disorders - anxiety about care. In this case, a higher level of anxiety predicts the onset of symptoms of postpartum depression. It can be assumed that concerning experiencing anxiety about care, there is no need to hide from the environment because caring for a child, also in social perception, is associated with numerous difficulties and challenges, so feeling uncertainty usually does not cause a negative assessment.

The other predictors, as in the MF group of mothers, refer to issues related to the child's feeding and include concern about infant undereating, awareness of infant's hunger and satiety cues, feeding infant on a schedule, and social interactions with the infant during feeding. The relationship between the first three and PPD is positive. It indicates that the greater the anxiety associated with various aspects of formula-feeding, the greater the risk of developing postpartum depression symptoms. It is worth noting that concern about infant undereating appeared in the regression model only in this group (FF group). Such results seem to complement the studies by Anato et al. (43), which indicate that maternal postpartum depression strongly correlates with inappropriate complementary feeding practices of infants and is a strong predictor of infants' undernutrition measured as stunting and underweight. The last predictor of postpartum depression in this group is poor interactions with the baby during feeding. This relationship, although noted in the current study, should, in our opinion, be interpreted with caution in order not to stigmatize formula-feeding mothers as having difficulties in establishing social interactions with the baby and further developing a mother-infant bond. The reason for this may be the presence of other factors that were not controlled in our study. At the same time, if the mother experiences difficulties in interacting with the baby during breastfeeding and sees the reasons for them in the fact that she feeds with bottles and formula milk, and the baby has no physical contact with the breast and breast milk, this may be a factor increasing the feeling of guilt over being an insufficiently good mother and thus aggravating the symptoms of PPD.

### Study Limitations and Implications for Further Research

Despite the high importance of identifying predictors of postpartum depression in terms of how the baby is fed in the first 6 months of life, our study has certain limitations that should be considered. First, the cross-sectional nature of the study precludes drawing causal conclusions. Thus, prospective longitudinal studies are needed to explore the association between postpartum depression symptoms and its potential risk among women in groups that differ in the feeding pattern. In addition, these studies should consider the relationship between breastfeeding intention and initiation when controlling for other PPD risk factors. Secondly, as participants were volunteers and the study sample was relatively small, especially when divided into different feeding types groups, and may not represent the total population. Thirdly, the limitations of the online survey as a data collection method (despite our various recruitment strategies) should also be mentioned, particularly sample bias. Those who, for various reasons, do not have access to the Internet, are not users of social media, or are unable to use information technology fluently cannot take part (44). Indeed, in our study, most women were well-educated, married, with satisfying economic situations, and living in large urban areas. This could call into question the generalizability of the findings. On the other hand, as Callegaro et al. (45) recommend, the possibility to complete the set of questionnaires in a safe Internet environment without any pressure may protect from social desirability bias. An additional limitation that should be taken into account is the low value of Cronbach's alpha coefficient for the IFQ factor describing the social interactions of the mother with the baby during feeding. A low value (α < 0.50) means low internal consistency, and thus may affect the reliability of the results obtained in the measurement for this factor. In spite of it, we included the obtained results because this factor appeared as a predictor only in the MF mothers group. However, we are aware of this limitation and its consequences. In further studies using IFQ, we recommend checking the factor structure of the questionnaire and, if necessary, determining factors with acceptable reliability for the tested sample. It should be noted as well that in most research EPDS is used usually by the 4^th^ month postpartum. Some women in our study filled in the EPDS at a later time, as our inclusion criteria considered delivery within the last 6 months. As EPDS Manual (46) presents evidence of the administration of the scale in the additional context (including women in the antenatal period and men) we decided to administer EPDS in our study even if more than usual time might have passed since delivery. As the scale was used as the indicator of depressive symptoms and not as the criterion for depression diagnosis the later time of its administration should not compromise its role as the screening tool. Finally, our study was not based on direct observation of mother-infant dyads during feeding episodes. Thus it relies on maternal self-reports related to feeding concerns and practices. Direct observation of maternal behavior in real feeding interactions might provide additional data on such concerns and possibly on predictors of depression. It is quite likely as a previous study (47) provided evidence on the difference in maternal-infant interactions between breastfeeding and bottle-feeding mothers.

Nevertheless, the study results indicate several vital relationships between postpartum depression symptoms among new mothers depending on how they feed their infants, which should be further investigated in prospective research.

## Conclusions

The differences in predictors of postpartum depression between three various types of infant feeding suggest that breastfeeding itself may not be a risk for postpartum depression. The specificity of maternal experiences with the various type of feeding is related to difficulties that can – in different ways – promote postpartum depression. Thus, providing emotional and educational support appropriate for different types of feeding may be an essential protective factor for postnatal depression. Participation in support groups for new mothers may facilitate the exchange of experiences and concerns and show women the similarity of their problems, while techniques adapted from cognitive-behavioral therapy (CBT) may help to manage maternal distress, what was already advocated for (48). Future investigation should focus not only on whether a woman breastfeeds but also on the importance of feeding methods for an individual and experiences related to feeding.

## Data Availability Statement

The raw data supporting the conclusions of this article will be made available by the authors, without undue reservation.

## Ethics Statement

The study was conducted according to the guidelines of the Declaration of Helsinki and approved by the university advisory board. As the study was of an informative cross-sectional purely descriptive nature, no formal ethical approval was required under the countries' legislations. Participants were informed of the purpose, risks, and benefits of the survey. They were told they could withdraw from the study at any time and for any reason and provided electronic informed consent. Electronic informed consent was obtained from all subjects involved in the study in accordance with the Ethics Guidelines for Internet-mediated Research by British Psychological Society.

## Author Contributions

KK and EB-B: conceptualization, resources, data curation, writing—original draft preparation, writing—review and editing, and supervision. KK: methodology, formal analysis, investigation, and project administration. All authors have read and agreed to the published version of the manuscript.

## Conflict of Interest

The authors declare that the research was conducted in the absence of any commercial or financial relationships that could be construed as a potential conflict of interest.

## Publisher's Note

All claims expressed in this article are solely those of the authors and do not necessarily represent those of their affiliated organizations, or those of the publisher, the editors and the reviewers. Any product that may be evaluated in this article, or claim that may be made by its manufacturer, is not guaranteed or endorsed by the publisher.

## Footnotes

^1^This criterion was set for the purpose of ensuring that mixed feeding or the formula feeding was not related to the introduction of complementary foods after 6 months. According to the WHO guidelines (1), up to the age of 6 months, the infant should be fed only with mother's milk.
